# Conformational and Intermolecular Interaction Analysis of Tiaprofenic Acid: A X-Ray Powder Diffraction and First Principle Modeling Analysis

**DOI:** 10.3390/molecules30173593

**Published:** 2025-09-02

**Authors:** Mattia Lopresti, Luca Palin, Marco Milanesio

**Affiliations:** 1 Dipartimento di Scienze e Innovazione Tecnologica, Università del Piemonte Orientale, Viale Teresa Michel 11, 15121 Alessandria, Italy; mattia.lopresti@uniupo.it (M.L.); luca.palin@uniupo.it (L.P.); 2 Nova Res S.r.l., Baluardo Partigiani 5, 28100 Novara, Italy

**Keywords:** tiaprofenic acid, NSAID, structure solution, Hirshfeld surface analysis, first principle calculations, X-ray powder diffraction

## Abstract

(±)-tiaprofenic acid (TA), marketed as (Surgam^®^), belongs to the family of NSAIDs, with the peculiarity of a reduced incidence of ulcer induction in rats compared with parent drugs. However, some adverse effects were observed, and better knowledge of its interaction with biologic substrates is needed. Unfortunately, unlike most commercial NSAIDs, suitable single crystals for an X-ray diffraction study could not be obtained. To fill the gap, the crystal structure of TA was solved by X-ray powder diffraction, and the molecular interactions stabilizing the structure were analyzed by Hirshfeld surface and energy framework analysis. TA crystallizes in the $P2_{1}/c$ space group, with its two enantiomers in the asymmetric unit, further confirming the peculiarity of the crystal structure and the difficulty of solving it. TA packing is characterized by alternating enantiomers connected through hydrogen bonds, forming chains, arranged in layers, stabilized by π-stacking. First principle modeling revealed several stable conformations within 4kJ ${\mathrm{mol}}^{-1}$ of the global minimum and the relaxed potential energy scans revealed modest (8kJ ${\mathrm{mol}}^{-1}$ to 15kJ ${\mathrm{mol}}^{-1}$) energy barriers. Such flat energy landscape suggests flexible and dynamic behavior of tiaprofenic acid in solution and in vivo conditions, with multiple suitable docking sites.

## 1. Introduction

Non-steroidal anti-inflammatory drugs (NSAIDs) have significantly impacted various aspects of human health. Their indications include, among others, the management of pain, inflammation, and fever in upper respiratory tract infections [1], as well as more specific clinical cases such as osteomyelitis [2], hip and knee arthroplasties [3], and postoperative pain following breast cancer surgery [4].

Ibuprofen was among the first NSAIDs to be commercialized, followed by the development and marketing of various other parent compounds [5]. Despite their therapeutic success, NSAID adverse effects have become increasingly evident [6]. Crystallographic studies on NSAIDs have contributed to understanding their molecular structures and possible interactions with biological targets [7,8,9]. Among these studies, powder X-ray diffraction has occasionally been employed when suitable single crystals could not be obtained [10,11].

Flanagan et al. [12] emphasized the critical importance of structural studies in investigating the interactions between NSAIDs and their molecular targets. Among these compounds, tiaprofenic acid (Surgam^®^) has shown effects on various human immune parameters, including decreased synthesis of both IgG and IgM [13]. Its effects on cartilage proteoglycans were first evaluated in rabbits [14], and later investigated in clinical trials related to arthroscopy [15] and molar surgery [16].

Notably, tiaprofenic acid (TA) showed a reduced incidence of ulcer induction in rats compared with other NSAIDs [17]. However, like other NSAIDs, TA presents some drawbacks, including photoreactivity [18], cystitis [19,20], and degradation-related issues [21]. As a chiral molecule, it can be obtained either as a racemic mixture or as a pure enantiomer [22].

Surprisingly, despite extensive clinical, chemical, and biological investigations, no crystal structure for TA has been reported in the literature or in crystallographic databases. This lack of structural data has hindered the development of docking studies, which have proven useful for other NSAIDs [23] in elucidating mechanisms of action and understanding the origins of side effects. Since TA is a flexible molecule, a crystal structure suggesting the stable conformation in the solid state and more stable intra and intermolecular interactions could be a critical starting point for conformational and docking studies, thereby better understanding its mechanism of action and the causes of adverse effects. In the case of TA, the absence of a known crystal structure has made it difficult to experimentally verify the stable conformers, especially due to its molecular flexibility. Computational methods struggle to reliably predict the most stable conformation of both enantiomers in either the solid state or in solution. This study aims to fill this gap by determining the stable molecular structure of TA in the solid state via X-ray powder diffraction, due to the inability to obtain suitable single crystals, supported by first-principle modeling to study in detail the stable conformations and the energy barriers among them. Molecular calculations were conducted to evaluate the energy penalty of the TA molecule when constrained within the crystal lattice and to shed light on the possible stable conformations when TA is in solution or in vivo conditions.

## 2. Results and Discussion

Understanding the solid-state structure of non-steroidal anti-inflammatory drugs (NSAIDs) is of paramount importance initially for ensuring proper pharmaceutical quality control. Crystal structure determination through X-ray diffraction enables detailed characterization of molecular packing, hydration states, and polymorphism, all of which influence key properties such as solubility, dissolution rate, and thermal behavior [24,25,26,27]. In this context, computational methods complement structural analysis by providing insight into phase transitions, energy landscapes, and conformational freedom, a key issue for bioactivity, and for proper docking within the drug target. Moreover, Hirshfeld surface analysis and related techniques allow us to understand the intermolecular interactions that promote crystal packing efficiency and stability. The following sections present the crystal structure of the racemic mixture of (±)-tiaprofenic acid (TA), with an emphasis on its molecular conformation, packing arrangement, the analysis of intermolecular interactions and the conformational analysis by first principle modeling.

### 2.1. Description of the Crystal Packing and Refinement

**Figure 1:**
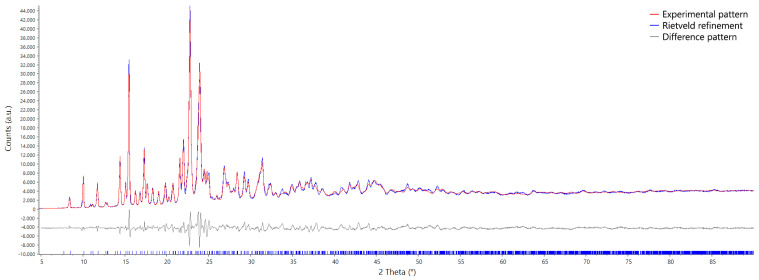
Rietveld refinement of the racemic crystal structure.

**Figure 2:**
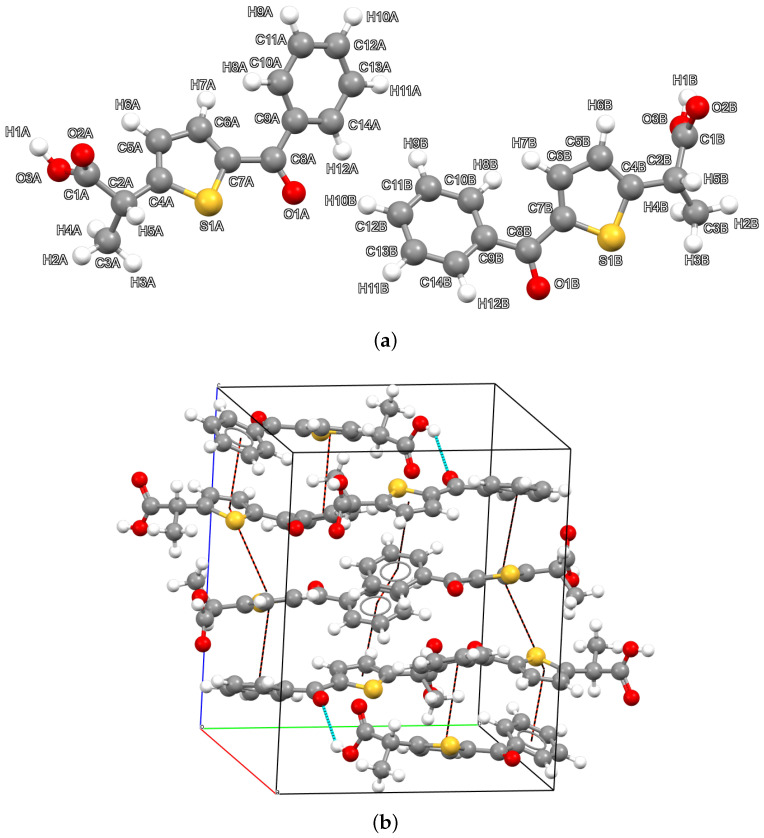
Asymmetric unit (**a**) and packing (**b**) of the title compound. In the asymmetric unit, label A was used for the atoms of the R enantiomer, while letter B was used for the atoms of the S enantiomer. In the crystal packing, intermolecular interactions are highlighted by red and light blue dotted lines for π and H-bond interactions, respectively.

The structure was solved by X-ray powder diffraction (XRPD), and a Rietveld refinement was performed, as detailed in the materials and methods section. Figure 1 shows the nearly perfect overlap between the experimental pattern (red curve) and the calculated pattern (blue curve). The difference pattern (grey curve) displays only minimal deviations, with very small spikes occurring at the positions of the main reflections. The apparent broadening of the peaks between 25° to 45° is due to the presence of bunches of adjacent peaks of similar intensity. No particular anisotropic broadening is suggested by the parameters obtained after the Rietveld refinements. TA crystallizes in the monoclinic system, within the $P2_{1}/c$ space group. The asymmetric unit (Figure 2a) is formed by the two enantiomers of TA in a 1:1 ratio. The presence of both optical isomers in the asymmetric unit allows for the crystallization of TA within a non-chiral space group. The unit cell parameters, reported along with other relevant crystallographic information in Table 1, are as follows: *a* = 11.435(2) Å, *b* = 13.625(3) Å, *c* = 16.180(3) Å, and β = 94.452(6)°. The crystal packing shown in Figure 2b reveals that TA crystallizes in sequences of alternating enantiomers connected through hydrogen bonds. Each R enantiomer is connected to the subsequent S enantiomer in the chain via the hydrogen bond O2A–H1A···O1B, and similarly, each S enantiomer is linked to the following R enantiomer through the O3B–H1B···O1A hydrogen bond. Such chains are arranged in layers parallel to the (0 0 l) family of planes. No hydrogen bonding is observed between adjacent chains. Despite the absence of directional interactions between the chains, the packing is compact and characterized by hydrophobic methyl and phenyl contacts, with a relatively low volume per atom of 17.45 ${Å}^{3}$/atom^−1^, and no voids are observed within the structure.

The packing, directed by hydrogen bonding, brings the thiophene and benzene rings of the R and S enantiomers, respectively, into close proximity across adjacent layers. The angle between the two ring planes is 13.57°, and the centroid-to-centroid distance is 3.767 Å, both consistent with weak π–π stacking interactions [28,29]. The displacement angle, calculated along the C10–Centroid(benzene)···Centroid(thiophene) vector, is 79.57°, corresponding to an angle of 10.43° between the centroid vector and the ring normal. This value falls within the range typically observed for parallel-displaced π–π stacking geometries, as described in Janiak’s analysis of aromatic interactions [30]. Within the crystal packing, the two torsional angles between the thiophene ring and the acid function, as well as between the thiophene and the phenyl ring, suggest that the two enantiomers, although adopting very similar side chain conformations, do not pack in an entirely identical fashion, because of the opposite orientation of H and CH_3_ on the chiral carbon. Specifically, the torsional angle $\tau_{C4A-C2A-C1A=O2A}$ in the R enantiomer is −90.46°, whereas the corresponding angle in the opposite enantiomer ($\tau_{C4B-C2B-C1B=O2B}$) is 96.62°. Likewise, the dihedral angle between the carbonyl group and the neighboring phenyl ring is 25.08° ($\tau_{O1A=C8A-C9A-C14A}$) for the R enantiomer and 27.33° ($\tau_{O1B=C8B-C9B-C14B}$) for the S enantiomer. Although these differences are relatively small, they are sufficient to highlight a slightly different chemical environment, which becomes evident in the subsequent Hirshfeld surface analysis and the associated fingerprint plots.

### 2.2. Hirshfeld Surface Analysis

**Figure 3:**
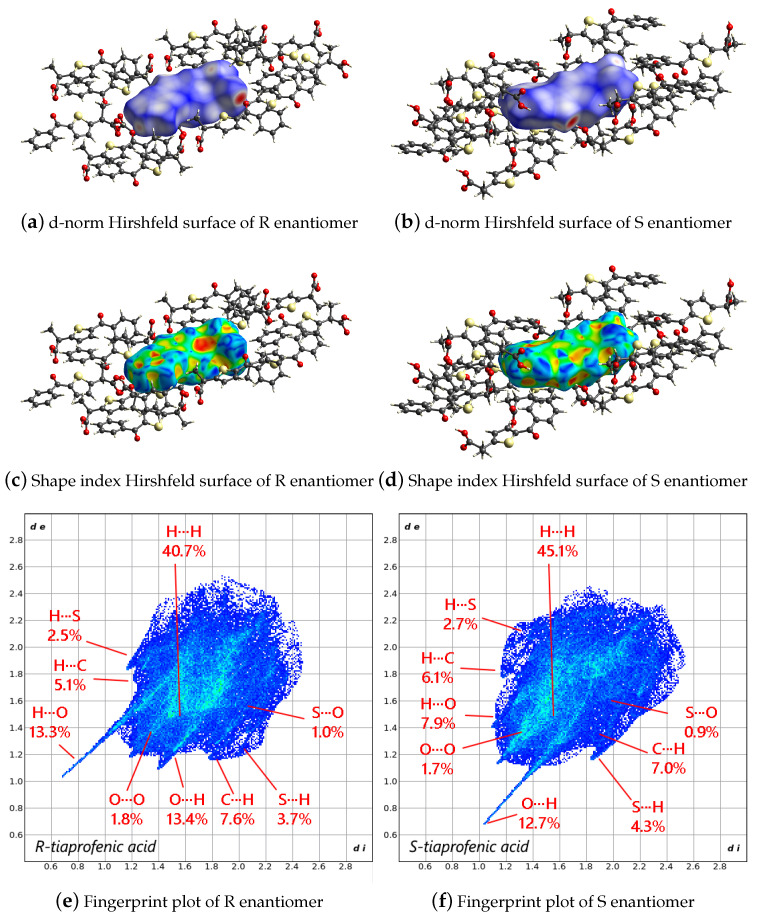
Hirshfeld surface highlighted for d-norm (**top**) and for the shape index (**center**), and the resulting fingerprint plots (**bottom**) for the two molecules of R-tiaprofenic (**left**) and S-tiaprofenic (**right**) acid in the asymmetric unit.

Hirshfeld surface analysis allows for a more detailed examination of the molecular packing described in the previous section. The surface mapped over *d_norm_* (i.e., the normalized contact distance; Figure 3a,b) exhibits predominantly blue and white regions, especially above and below the enantiomers, corresponding to the faces parallel to the planes of the hydrogen bonds that connect one enantiomer to the next. This coloration indicates that the intermolecular contacts between these molecular planes are either close to (white) or longer than (blue) the sum of the van der Waals radii. Shorter contacts are highlighted in light red on the surface and are mainly observed near the carboxylic acid groups, indicating weak interactions between adjacent layers. In particular, a close contact is detected between hydrogen atom H4B of the methyl group in the S-enantiomer and oxygen atom O2A of the carboxylic acid in the R-enantiomer. Within the planes composed of alternating R and S enantiomers, shorter contacts correspond to the hydrogen bonds that form the one-dimensional chains. Such minor features underscore that the local chemical environments of the two enantiomers are not identical. When the surface is mapped over the shape index (Figure 3c,d), a qualitative descriptor of surface curvature sensitive to subtle variations, especially in regions of low overall curvature, weak π–π interactions become evident. Specifically, a characteristic red triangle at the center of the phenyl ring of the R-enantiomer, accompanied by a yellow-green halo on its sides, indicates a complementary interaction with the thiophene ring of the S-enantiomer. The matching complementary shape (a blue triangle with a blue-green halo) appears on the surface of the thiophene ring of the S-enantiomer, confirming that weak π–π interactions help stabilize the stacking of the alternating R–S molecular layers. A similar pattern is observed for the thiophene ring of the R-enantiomer and the phenyl ring of the S-enantiomer. The fingerprint plots presented in Figure 3e,f, corresponding to the TA enantiomers R and S, illustrate a closely comparable, almost specular, but not identical, distribution of intermolecular contacts. These subtle differences breaking the perfect mirroring, expected if R and S enantiomers had identical symmetrical environments, primarily stem from variations in three key torsional angles: (i) the conformation around the carboxylic moiety, namely, the rotation involving carbonyl oxygen and the methylene bridge connecting to the thiophene ring; (ii) the relative orientation of the methyl group of the 2-carboxypropyl side chain with respect to the thiophene plane and (iii) the inclination of the carbonyl group attached to the phenyl ring. Although the overall molecular geometries remain very similar, these torsional deviations (quantified to vary between 0.1% and 4.4% in their contribution to the Hirshfeld surface contacts) are sufficient to alter the local interaction patterns. The non-mirrored contact maps reflect the fact that each enantiomer, despite adopting a comparable conformation, is embedded in a distinct chemical environment within the crystal lattice. This arises from the alternating R–S stacking, which is mediated by directional hydrogen bonding and further stabilized by weak π–π interactions involving the phenyl and thiophene rings. The fingerprint plots thus provide a detailed representation of how even modest conformational asymmetries can propagate into measurable differences in the molecular packing landscape. These findings underscore the importance of detailed stereochemical and conformational analysis when evaluating racemic crystals, as enantiomeric environments can diverge even under overall symmetric packing conditions, as in the solved crystal structure. To further investigate the conformational freedom of TA, a detailed geometric and energetic first-principles analysis of the isolated molecule is performed, and the results are presented in the following sections.

### 2.3. Energetic Considerations

The solved crystal structure represents a solid starting point for modeling TA to understand the energy landscape of the molecule. At first, a conformational analysis is carried out to understand the flexibility of the molecule and the possible interconversion among the stable conformers. Finally, the energetic analysis of the crystal packing is investigated using the energy framework approach and and the results are compared with the data obtained by the modeling of the isolated molecule.

#### 2.3.1. Conformational Analysis on the Isolated Molecule

The R and S enantiomers in the asymmetric unit of the crystal structure exhibit similar orientations of the side chain but opposite configurations of the hydrogen and methyl substituents on the C2 stereocenter (atom labels as in Figure 2a). Specifically, the carboxylic group adopts an anti conformation with respect to the C2 substituents along the C2–C1 bond. Similarly, the carboxylic OH group is oriented anti to the O3–C1 bond, optimizing intermolecular hydrogen bonding. At the same time, the methyl hydrogen atoms are staggered relative to the neighboring C2–H5 bond, minimizing intramolecular steric hindrance.

In contrast, the 2-carboxypropyl and phenyl moieties adopt significantly different conformations in the R and S enantiomers. In the R enantiomer, the 2-carboxypropyl group adopts a more staggered conformation. Although the C14–C9–C8=O1 torsion angle shares the same sign in both enantiomers, its spatial orientation relative to the thiophenic ring differs, with the S enantiomer appearing more planar.

The molecular structure suggests four main degrees of conformational freedom: three associated with the 2-carboxypropyl chain (C1–C2, C2–C4 and C2–O3) and one governing the phenyl orientation (C9–C14). The torsions around C1–C2 and C2–C4 are adjacent and in close relationship. In contrast, the orientations of the carboxylic OH are determined by the crystal environment (through hydrogen bonding and π-stacking interactions, respectively) and are less directly related to the main chain conformation. The methyl orientation (reported in Table 2 for sake of completeness) is clearly not relevant for overall molecular stability and thus not included in PES analyses.

After these considerations, three series of calculations were performed. First, the R and S conformations determined by XRPD were relaxed, optimizing all geometrical parameters while keeping the four key torsion angles fixed to their experimental solid-state values. This approach was used to estimate the energetic cost of the conformations imposed by the crystal lattice constraints with respect to the isolated molecule calculations.

**Figure 4:**
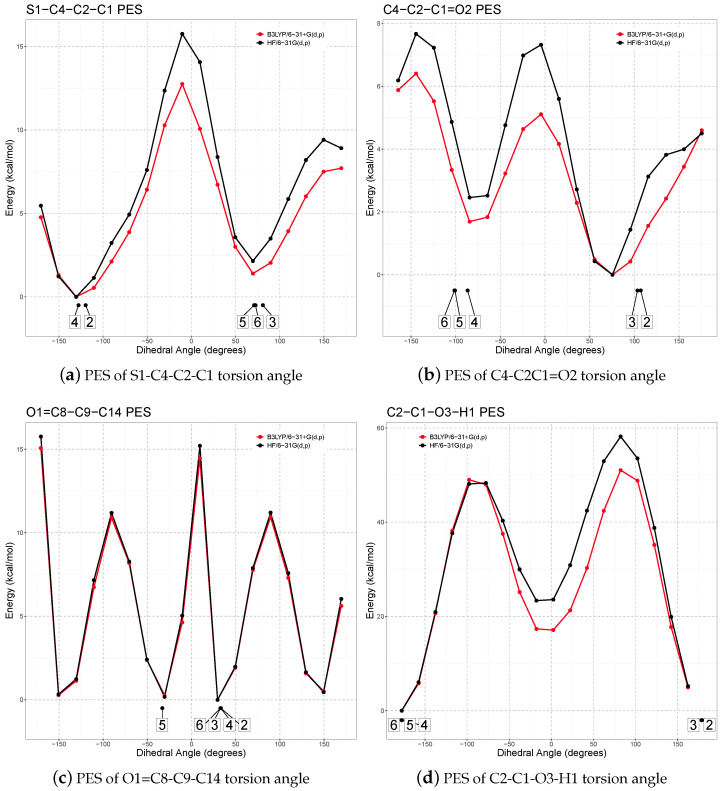
HF and B3LYP 1D PES on the 4 degrees of freedom for the S enantiomer; the position of the stable conformers after B3LYP/6-31+G(d,p) geometry optimization is highlighted.

Then, two families of potential energy surface (PES) scans were performed. Four one-dimensional (1D) PES scans (Figure 4) were computed for the torsion angles identified above, which control the orientations of the side chain and phenyl ring with respect to the rigid, planar thiophene moiety. These calculations were carried out at both a low level of theory (HF/6-31G(d,p)) and a higher level (B3LYP/6-31+G(d,p)) to compare accuracy and computational cost.

**Figure 5:**
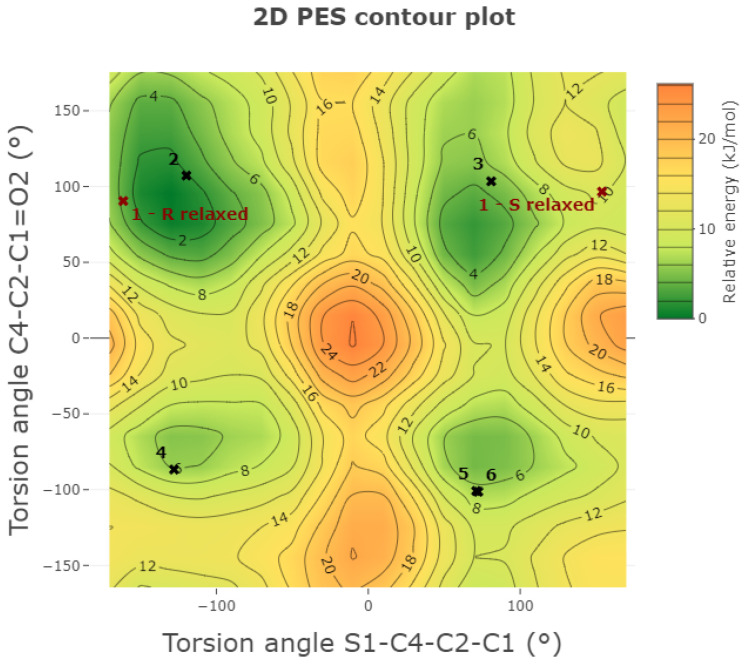
2D PES generated to explore the side chain flexibility; S1–C4–C2–C1 and C4–C2–C1=O3 torsion angles were scanned with a 20° step.

Additionally, a two-dimensional (2D) PES scan (Figure 5) was carried out at the HF/6-31G(d,p) level. This level was chosen to make the large number of required calculations feasible. The 2D scan simultaneously explored the C1–C2 and C2–C4 torsion angles, which are adjacent and conformationally coupled, to investigate side chain flexibility, identify stable conformers, and determine the energy barriers between them. All unique conformers identified from the PES scans were then fully optimized at the B3LYP/6-31+G(d,p) level of theory to assess their relative stabilities. The corresponding geometric and energetic data are summarized in Table 2.

Analysis of the 1D and 2D PESs revealed two energy minima for each side chain torsion angle. When combined with the two possible orientations of the phenyl ring, a total of 16 theoretical conformers (8 for each enantiomer) were generated and subsequently optimized. One phenyl ring orientation was found to be consistently more stable, corresponding to a positive O1=C8–C9–C14 torsion angle in the S enantiomer and a negative value in the R enantiomer. Three out of the eight generated conformers converged to conformers 2, 3, or 4 upon optimization.

In total, five independent stable minima were identified and added to the XRPD-derived R conformer, yielding six low-energy conformations for each enantiomer. Ten distinct conformers (five R and five S) were found within 4 kJ ${\mathrm{mol}}^{-1}$ of the global minimum, highlighting the high conformational flexibility of TA.

Furthermore, the calculated energy barriers between these conformers ranged from 8kJ ${\mathrm{mol}}^{-1}$–15kJ ${\mathrm{mol}}^{-1}$, values that are accessible at room temperature. This suggests a highly dynamic conformational landscape for TA in solution and under in vivo conditions, enabling it to adapt its geometry to different biological environments and potentially engage multiple binding sites.

The stable conformations obtained by first-principle calculations differ from those observed in the experimental XRPD structures. Notably, the conformational differences between R and S enantiomers in the crystal structure are confirmed in the gas-phase analysis. The relaxed 1-S conformation closely resembles conformation 3, but it lies far from the global minimum, with an energy penalty of 8.4 kJ ${\mathrm{mol}}^{-1}$, as evident from its position on the 2D PES scan.

Conversely, the relaxed 1-R conformation does not match any of the identified stable conformers, as highlighted by the torsion angles reported in Table 2. While its side chain resembles that of conformation 2 (as seen in the 2D PES), the phenyl ring is rotated into a less favorable orientation, similar to that in conformation 5. This specific combination, not observed in any isolated low-energy conformer, results in an energy penalty of 10.9 kJ ${\mathrm{mol}}^{-1}$.

These elevated energy values indicate that the experimental conformations are stabilized not by intrinsic molecular geometry but by favorable intermolecular interactions, particularly hydrogen bonding and crystal packing effects. This highlights the remarkable conformational adaptability of tiaprofenic acid, which is likely critical for its biological activity and interaction with various molecular targets [31].

#### 2.3.2. Energy Frameworks Analysis and Lattice Energy Calculation

**Figure 6:**
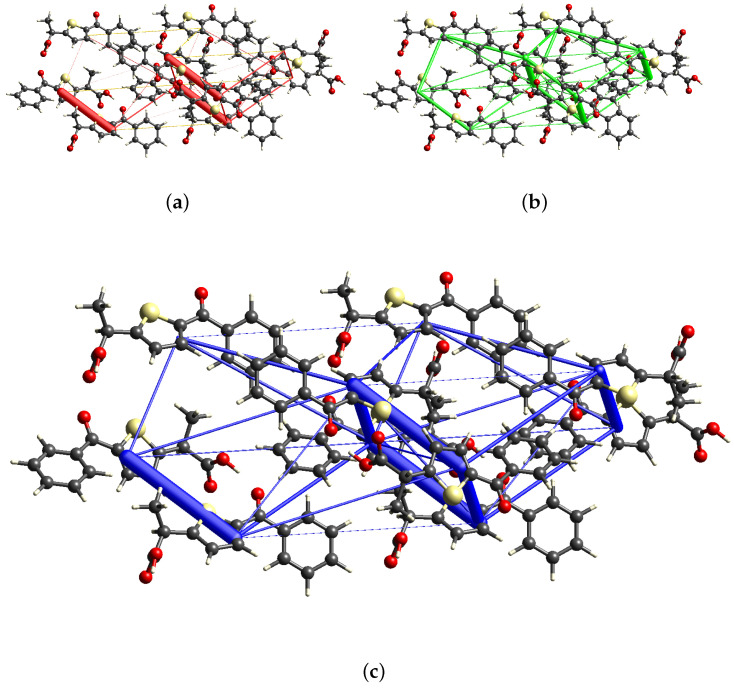
Energy framework calculated for the title compound. (**a**): Coulomb energy; (**b**): dispersion energy; (**c**): total energy.

Figure 6 shows the energy frameworks calculated for the TA crystal structure using CrystalExplorer, with the 6-31+G(d,p) 5D basis set and the B3LYP level of theory. The dominant contribution to the framework comes from Coulombic interactions (represented by red cylinders in Figure 6a), which are primarily oriented along the direction of the alternating chains of enantiomers. A smaller but non-negligible stabilizing contribution is provided by dispersion interactions (green cylinders in Figure 6b), which occur both between adjacent chains and along the same direction as the enantiomer chains. Such dispersion interactions are particularly relevant in the hydrophobic regions of the crystal, where polar contacts are absent. Their presence supports previous structural observations indicating the importance of van der Waals contacts in these areas. It is worth noting the comparison of such indications by energy frameworks with the crystal packing features. The larger and dominant red cylinders in Figure 6a are perfectly aligned along the hydrogen bond direction of the crystal structure (light blue lines in Figure 2b direction. Conversely, the net of green cylinders in Figure 6b is more complex without a dominant direction, as explained by the different π-π interactions evidenced by red lines in Figure 2b, i.e., the thiophene/thiophene and phenyl-phenyl contacts Figure 2b. However, the observed spatial distribution of the total energy (blue cylinders in Figure 6, representing the vectorial sum of Coulombic and dispersion contributions helps to rationalize the compactness of the crystal structure, due to the above-described mixture of Coulombic and dispersion interactions. The calculated lattice energies are −127.7 kJ ${\mathrm{mol}}^{-1}$ and −145.0 kJ ${\mathrm{mol}}^{-1}$ for the R and S enantiomers, respectively. This value largely exceeds the conformational penalty calculated for the R and S conformation (10.9 kJ ${\mathrm{mol}}^{-1}$ and 8.4 kJ ${\mathrm{mol}}^{-1}$ in Table 2), also considering the multiplicity of the unit cell containing 4 R and 4 S enantiomers. This situation confirms that the energy gain of the crystal structure is due to the packing, much more than the internal molecular R and S conformations, different from the absolute minimum for the isolated molecule (conformer 4 in Table 2). We can conclude that TA adapts its molecules’ conformation to the solid state environment to reach the minimum possible total lattice energy. Because of the relatively flat energetic landscape (Figure 5), we can envisage a similar behavior also in solution and in vivo when interacting with biological substrates and pharmacological targets.

## 3. Materials and Methods

(±)-tiaprofenic acid (TA) was purchased from Sigma-Aldrich (now Merck KGaA, Darmstadt, Germany) (98% purity certified by the supplier). Since a conformal chemical analysis is guaranteed by the supplier, the product was used without further purification. Recrystallization efforts were performed with the same approach as in previous works [32,33], but no crystals suitable for single-crystal X-ray diffraction were obtained. The sample was, therefore, collected directly from the bottle and gently ground before analysis by X-ray powder diffraction (XRPD), obtaining a white homogeneous powder with a crystal size smaller than 5 microns, assessed by optical microscopy examination.

### 3.1. XRPD Characterization and Refinement

XRPD data of TA were collected using a Bruker D8 Advance powder diffractometer equipped with a Cu $K_{\alpha }$ radiation source [34] and a LynxEye XE-T detector operating in 1-D mode. The source was set at 40 mA current and 40 kV electric potential. The radius of the goniometer was set to standard operating conditions at 280 mm. Soller slits with an angle of 2.5° were mounted on both primary and secondary optics to correct for the axial divergence. The sample was gently ground and placed in a standard polycarbonate sample holder. In primary optics, variable width slits were mounted and set to constantly irradiate the width portion of the 17 mm width sample at every angle. The secondary path was kept free of optics, except for the Soller slits. Data collection was carried out in Bragg–Brentano geometry in the 2θ range of 4° to 90° angle with a step size of 0.02° ${\mathrm{step}}^{-1}$ and a collection time of 1 s ${\mathrm{step}}^{-1}$. The indexing procedure was carried out using the N-TREOR09 [35] routine included in EXPO2014 [36], and the space group was then selected based on agreement factors and minimization of non-indexed reflections. The structure solution was performed with EXPO2014 using the simulated annealing method in direct space. The final Rietveld refinement has been performed using TOPAS-Academic V7 [37,38]. The copper tube radiation was modeled according to the official NIST copper $K_{\alpha }$ spectrum high-precision measurement of the X-ray Cu $K_{\alpha }$ spectrum [34]. A rigid body for each molecule in the asymmetric unit was used to obtain a reliable data/parameter ratio. The preferred orientation effects were modeled using spherical harmonics, and the peak profile was refined using a pseudo-Voigt function. For non-H atoms, a global thermal parameter for each molecule was refined, as is usually conducted in powder diffraction data, because of the well-known problem of the poor data/parameter ratio. Final values of 0.026(2) and 0.028(2) were obtained for the thermal parameters of the two TA molecules. Structure visualization and image generation were performed using CCDC Mercury [39]. Crystallographic data were submitted to the CCDC database, and the 2477873 CCDC number was assigned. The XRPD pattern and relative indexing information were submitted to ICCD [40].

### 3.2. In Silico Analyses

The R and S XRPD geometries were relaxed by Gaussian 16 [41], using the density functional theory (DFT) method with the B3LYP level of theory and the 6-31+G(d,p) basis set with 5d option. The conformational freedom of the torsion angles of the main degrees of freedom was explored by potential energy scans (PES) at the HF/6-31G(d,p) level of theory. This approach was used because a 2D PES requires $18^{2}$ geometry optimizations. To validate the HF/6-31G(d,p) 2D PES, 1D PESs around the main degrees of freedom, governing the side chain, phenyl and OH orientations, were calculated at this level of theory and compared with the result obtained using B3LYP/6-31+G(d,p). All PESs were calculated with a step of 20°, relaxing the structure at each value of the selected torsion angle. Since both methods identified the same energy minima, as detailed in the Results section, the conformations predicted from the 1D and 2D PES scans were generated starting from conformation 1-S (i.e., the S enantiomer of the crystal structure), labeled from 2 onward and optimized at the B3LYP/6-31+G(d,p) level of theory. Then, the corresponding R conformations were generated and their geometries optimized by the same approach. The Hirshfeld surfaces, the resulting fingerprint plots, and energy frameworks were obtained through first-principles calculations using CrystalExplorer 21.5 [42,43]. Hirshfeld surfaces were calculated in high-resolution settings. The wave functions of the two molecules in the asymmetric unit were determined using Gaussian 16 at the B3LYP level of theory with the 6-31+G(d,p) basis set, which is consistent with the geometric optimization methods. For the figures of the energy framework, the tube size scale was set to 80 (default) and the energy cutoff value was 0 kJ ${\mathrm{mol}}^{-1}$.

## 4. Conclusions

The crystal structure analysis of (±)-tiaprofenic acid (TA) revealed several noteworthy features. Most significantly, two enantiomeric molecules are present in the asymmetric unit, a non-standard arrangement that likely contributes to the difficulty in growing suitable single crystals and explains the lack of structural data in the literature. This configuration also highlights strong interactions between enantiomers, forming tightly hydrogen-bonded chains. Such supramolecular organization suggests that intermolecular forces play an important role in stabilizing the solid-state structure and may influence molecular behavior under biological conditions. The rather high propensity of TA towards H-bond interaction was already suggested by Van Duong et al. [44] by a careful analysis of the FTIR spectra.

First principle modeling of the isolated molecule identified six distinct low-energy conformations per enantiomer (one from XRPD and five calculated), all within 4 kJ ${\mathrm{mol}}^{-1}$ of the global minimum. These conformations are separated by relatively low energy barriers (8kJ ${\mathrm{mol}}^{-1}$–15kJ ${\mathrm{mol}}^{-1}$), indicating a highly dynamic conformational landscape in solution and potentially under in vivo conditions. Such low barriers suggest that TA can readily interconvert between conformations and adapt its geometry to fit the surrounding molecular environment.

This adaptability is further supported by the crystal packing analysis. The experimentally observed R and S conformers differ from the most stable gas-phase structures due to constraints imposed by the crystal lattice. This confirms that intermolecular interactions (particularly hydrogen bonding and π-stacking highlighted by energy framework calculations) can override intrinsic conformational preferences (suggested by molecular calculations).

However, further experimental work is needed to determine whether these structural and conformational differences influence the biological activity of the two enantiomers. Overall, the combined insights from crystal structure determination and first-principles modeling provide a detailed picture of TA’s conformational flexibility and intermolecular behaviors. These findings offer a valuable foundation for future docking studies aimed at elucidating TA’s mechanism of action and guiding the design of new derivatives with improved therapeutic profiles and reduced side effects.

## Figures and Tables

**Table 1:** Crystal structure and final Rietveld refinement parameters for the title compound.

Name	(±)-Tiaprofenic Acid
Empirical Formula	C_14_ H_12_ O_3_ S
Formula weight/$\;g\;{\mathrm{mol}}^{-1}\;$	260.30
Temperature/K	293(2)
Crystal system	monoclinic
Space group	$P2_{1}/c$
a/Å	11.435(2)
b/Å	13.625(3)
c/Å	16.180(3)
α/ °	90
β/ °	94.452(6)
γ/ °	90
Volume/${Å}^{3}\;$	2513.3(8)
Average atomic volume/${Å}^{3}\;$	17.45
Z	8
$\rho_{calc}$ $g\;{cm}^{-3}\;$	1.376
µ$/{mm}^{-1}\;$	2.275
F(000)	1088.0
Radiation/Å	$\begin{array}{c} Cu \end{array}$ $K_{\alpha }$
2θ range for data collection/°	4 to 90
Reflections used for indexing	36
Rp/Rwp	5.390/7.461

**Table 2:** Geometric and energy features of the conformations obtained by XRPD and after B3LYP/6-31+G(d,p) geometry optimization (relaxed), together with the ones obtained analyzing the 1D e 2D PES.

Chirality	Torsion Angle	S1–C4–C2–C1	C4–C2–C1=O2	O1=C8–C9–C14	C2–C1–O3–H1	H5–C2–C3–H3	Relative Energy
	Conformation	(°)	(°)	(°)	(°)	(°)	($kJ\;{\mathrm{mol}}^{-1}\;)$
S	1-S relaxed	153.9	96.6	27.3	−180.0	58.8	8.4
S	2	−119.7	107.2	33.2	178.9	−58.8	4.1
S	3	80.9	103.5	32.8	178.3	−58.5	3.0
S	4	−127.8	−86.7	33.0	−177.5	−56.6	0.1
S	5	71.0	−100.9	−33.1	−177.6	−56.9	1.8
S	6	72.5	−101.4	32.7	−177.9	−57.0	1.5
R	1-R relaxed	161.3	−90.5	25.1	−170.0	−59.2	10.9
R	2	119.6	−106.1	−33.2	−178.9	−60.6	3.8
R	3	−77.8	−104.8	−32.7	−178.3	−60.9	3.2
R	4	126.9	87.5	−33.0	177.4	−63.3	0.0
R	5	−71.0	100.9	33.1	177.6	−63.1	1.8
R	6	−71.0	100.9	-33.1	177.6	57.0	1.5

R and S relaxed blocking C1–C2, C2–C4, C2–O3 and C8–C9 torsion angles.

## Data Availability

Data will be made available on request. CCDC 2477873 contains the supplementary crystallographic data for the title compound. These data can be obtained free of charge from https://www.ccdc.cam.ac.uk/, 29 August 2025 or from the Cambridge Crystallographic Data Centre, 12 Union Road, Cambridge CB2 1EZ, UK; fax: (+44) 1223-336-033; or e-mail: deposit@ccdc.cam.ac.uk. Crystallographic information file and XRPD pattern data and refinement were submitted to ICDD.
